# LncRNA WTAPP1 promotes cancer cell invasion and migration in NSCLC by downregulating lncRNA HAND2-AS1

**DOI:** 10.1186/s12890-020-01180-0

**Published:** 2020-05-30

**Authors:** Li Zhang, Chengyan Jin, Guiyun Yang, Bin Wang, Peiyan Hua, Yan Zhang

**Affiliations:** 1 Department of Anesthesiology, The Second Hospital of Jilin University, Changchun City, Jilin Province 130041 People’s Republic of China; 2 Department of Thoracic Surgery, The Second Hospital of Jilin University, No.218 Ziqiang Street, Changchun City, Jilin Province 130041 People’s Republic of China; 3 Department of Operating Room, The Second Hospital of Jilin University, Changchun City, Jilin Province 130041 People’s Republic of China

**Keywords:** Non-small cell lung cancer, lncRNA WTAPP1, lncRNA HAND2-AS1, Survival

## Abstract

**Background:**

Long non-coding RNA (lncRNA) Wilms Tumor 1 Associated Protein Pseudogene 1 (WTAPP1) has been reported to be a critical player in the angiogenesis and migration of endothelial progenitor cells, while its involvement in cancer biology remains unknown. This study was carried out to investigate the role of WTAPP1 in non-small cell lung cancer (NSCLC).

**Methods:**

The expression of WTAPP1 and lncRNA HAND2 Antisense RNA 1 (HAND2-AS1) in plasma and tissues from NSCLC patients was detected by qRT-PCR. A 5-year follow-up study was carried out to explore the prognostic value of WTAPP1 for NSCLC. Overexpression experiments were performed to analyze the interaction between WTAPP1 and HAND2-AS1. Cell invasion and migration were evaluated by Transwell assays.

**Results:**

The expression of WTAPP1 was upregulated in NSCLC. The survival analysis showed that low plasma levels of WTAPP1 were accompanied with high survival rate. HAND2-AS1 was downregulated in NSCLC and inversely correlated with WTAPP1 across tumor tissues. Overexpression of WTAPP1 resulted in downregulation of HAND2-AS1 in NSCLC cells, while overexpression of HAND2-AS1 did not affect the expression of WTAPP1. Overexpression of WTAPP1 led to promoted, while overexpression of HAND2-AS1 resulted in inhibited invasion and migration of NSCLC cells. In addition, overexpression of HAND2-AS1 partially attenuated the effects of overexpressing WTAPP1. In addition, WTAPP1 did not affect cancer cell proliferation.

**Conclusion:**

WTAPP1 may promote cancer cell invasion and migration in NSCLC by downregulating lncRNA HAND2-AS1.

## Background

Lung cancer affected 2,093,876 new cases (11.6 of all cancer patients) and caused 1,761,007 deaths (18.4% of all cancer deaths) in 2018 alone [1]. Lung cancer is the most common cancer for both incidence and mortality in many countries of the world [2] including China [3]. Lung cancer patients diagnosed before tumor invasions can be treated with surgical resections, and the treatment outcomes are generally satisfactory [4]. However, most patients with lung cancer are diagnosed at late stages with the existing of tumor metastasis and radical treatment approaches are not available [5].

Analysis of human transcriptome has revealed that the majority of RNA transcripts are not related to protein synthesis [6]. Long (> 200 nt) noncoding RNAs (lncRNAs) have structural and regulatory functions. Different from messenger RNAs that are templates of protein synthesis, lncRNAs function by affecting gene expression at different levels [7]. Altered expression of certain lncRNAs may mediate the aberrant expression of genes in human diseases including cancer, and regulation of lncRNAs can assist cancer treatment [8, 9]. However, the functions of most lncRNAs are still barely known. LncRNA WTAPP1 promotes angiogenesis and migration of endothelial progenitor cells [10], while contribute to cancer development [11]. Our study was therefore carried out to investigate the role of WTAPP1 in non-small cell lung cancer (NSCLC).

## Methods

### Research subjects

A total of 68 patients with NSCLC were enrolled in the Second Hospital of Jilin University from January 2011 to August 2018. Histopathological examination was performed to confirm the cancer. Inclusion criteria: 1) NSCLC patients were willing to receive biopsy; 2) newly diagnosed cases; 3) no therapy was performed within 3 months before admission. Exclusion criteria: 1) patients were suffering from multiple diseases; 2) patients initiated therapy. The 68 patients include 40 males and 28 females. The age range was from 30 to 69 years old, and the mean age was 50.6 ± 7.0 years old. Based on AJCC staging, there were 12, 14, 18 and 24 cases at stage I-IV, respectively. This study was approved by the Ethics Committee of the Second Hospital of Jilin University approved before the admission of patients. All patients signed the informed consent.

### Tissue specimens and cell lines

All patients received lung biopsy, and the tumor tissues and adjacent healthy tissues (about 0.1 g) were obtained from each patient. NSCLC cell lines H1581 and H1993 (ATCC, USA) were used. H1581 cell line was from a 44 years old male and H1993 cell line was from a 47 years old female. These two cell lines were used to match the patients. The cell culture medium was composed of 90% RPMI-1640 medium and 10% fetal bovine serum (FBS). Cells were cultivated at 37 °C with 5% CO_2_.

### Follow-up study

Patients were monitored for 5 years after admission through outpatient visit or telephone once a month. Patients who lost to follow-up or died of causes unrelated to NSCLC were not included.

### Total RNAs extracion and real-time quantitative PCR (qRT-PCR)

Total RNAs were isolated from tissue specimens and cells using RNAzol reagent (Sigma-Aldirch). The qPCR reactions were prepared using the SYBR® Green Master Mix (Toyobo, Japan). The expression levels of WTAPP1 and HAND2-AS1 were determined with 18S RNA as endogenous control. Ct values were normalized according to 2^-ΔΔCT^ method.

### Cell transfections

Full length of WTAPP1 and HAND2-AS1 genomic DNAs were cloned into pcDNA3.1 vector to establish WTAPP1 and HAND2-AS1 expression vectors. The vector constructions were performed by Sangon (Shanghai, China). Vector (10 nM) was transfected into cells using Lipofectamine 2000 reagent (Invitrogen). Cells transfected with empty vector were used as negative control (NC). Un-transfected cells cultivated under normal conditions until the end of transfections were used as the control (C).

### Cell migration and invasion assays

Transwell assays were performed at 24 h after transfection. Cells were transferred to the upper chamer with 3000 cells in 0.1 ml serum-free medium per well. Matrigel (Millipore, USA)-coated membranes were used in invasion assay, and uncoated membranes were used to carry out migration assay. In both assays, the lower Transwell chamber was filled with medium supplemented with 20% FBS. Cells were cultured for 2 h. After that, membrane was collected, cleaned and subjected to 0.5% crystal violet (Sigma-Aldrich) staining at room temperature for 14 min. Cells were evaluated under an optical microscope.

### Statistical analysis

Data were expressed with the mean ± standard deviation (SD) from 3 biological replicates. Graphpad Prism 6 software was used for all data analysis. Paired t test was used to compare differences between two groups. ANOVA Tukey’s test was used to compare differences among multiple groups. Linear regression analysis was carried out to explore the correlation between WTAPP1 and HAND2-AS1. According to Youden’s index, patients were divided into high (*n* = 36) and low (*n* = 32) WTAPP1 expression (tumor tissue) group. Survival curves were plotted and compared by log-rank test. Logistic regression was performed to identify the independent risk factor for NSCLC. Differences with *p* < 0.05 were considered as statistically significant.

## Results

### WTAPP1 was overexpressed in tumor tissues and correlated with survival

**Fig. 1:**
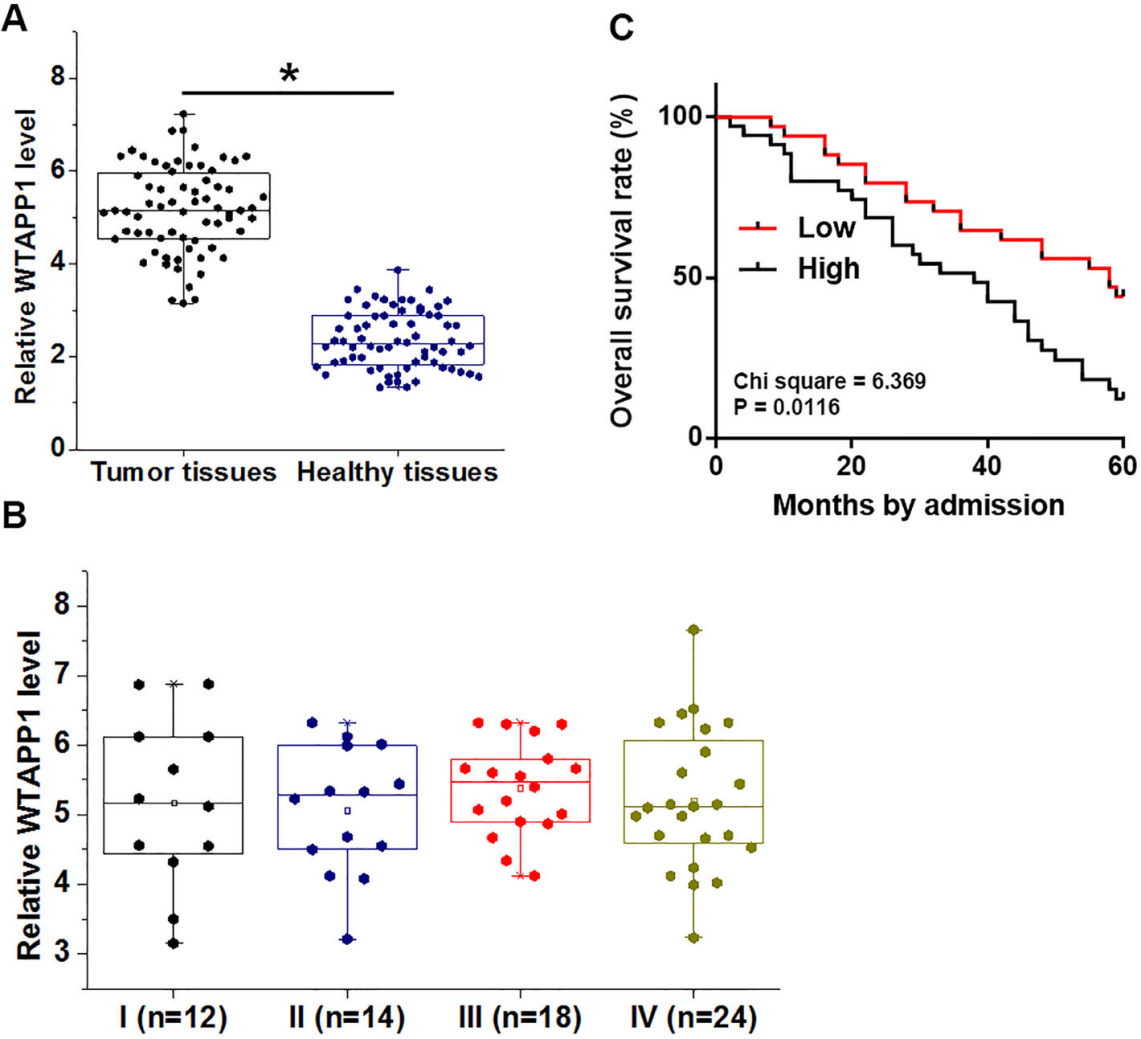
WTAPP1 was overexpressed in tumor tissues and correlated with survival. RT-qPCR revealed that WTAPP1 was overexpressed in tumor tissues than in adjacent healthy tissues **a**, (*, *p* < 0.05), and the expression of WTAPP1 in tumor tissues was not significantly affected by clinical stages **b**. Survival curve analysis showed that low plasma levels of WTAPP1 were closely correlated with high overall survival rate **c**

RT-qPCR was performed to investigate the differential expression of WTAPP1 in 68 NSCLC patients. Compared to healthy tissues, expression levels of WTAPP1 were significantly higher in tumor tissues (Fig. 1a, *p* < 0.05). However, clinical stages did not affect the expression levels of WTAPP1 (Fig. 1b). According to Youden’s index, patients were divided into high (*n* = 36) and low (*n* = 32) WTAPP1 expression (tumor tissue) groups, compared to patients in the low WTAPP1 expression level group, patients in the high WTAPP1 expression group level showed significantly lower overall survival rate (Fig. 1c). Logistic regression showed that high expression levels of WTAPP1 is an independent risk factor for NSCLC (odds ratio = 1.673; 95% confidence interval: 1.384 ~ 1.765; *p* = 0.024).

### HAND2-AS1 was inversely correlated with WTAPP1 in tumor tissues

**Fig. 2:**
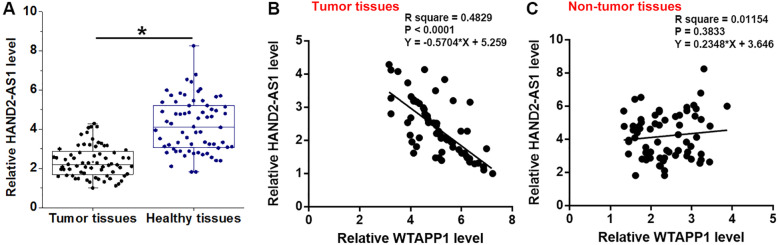
LncRNA HAND2-AS1 was downregulated in tumor tissues and was inversely correlated with WTAPP1. RT-qPCR revealed that HAND2-AS1 was downregulated in tumor tissues than in adjacent healthy tissues **a**, (*, *p* < 0.05). Linear regression showed that HAND2-AS1 was inversely correlated with WTAPP1 expression only in tumor tissues **b**, but not in adjacent healthy tissues **c**

Differential expression of HAND2-AS1 in 68 NSCLC patients was also detected. Compared to healthy tissues, the expression levels of HAND2-AS1 were significantly lower in tumor tissues (Fig. 2a, *p* < 0.05). Linear regression was used to analyze the correlation between WTAPP1 and HAND2-AS1. It was observed that the expression of HAND2-AS1 in tumor tissues was significantly correlated with the expression of WTAPP1 (Fig. 2b). However, WTAPP1 and HAND2-AS1 in adjacent healthy tissues were not significantly correlated.

### Overexpression of WTAPP1 mediated the downregulation of HAND2-AS1 in NSCLC cells

**Fig. 3:**
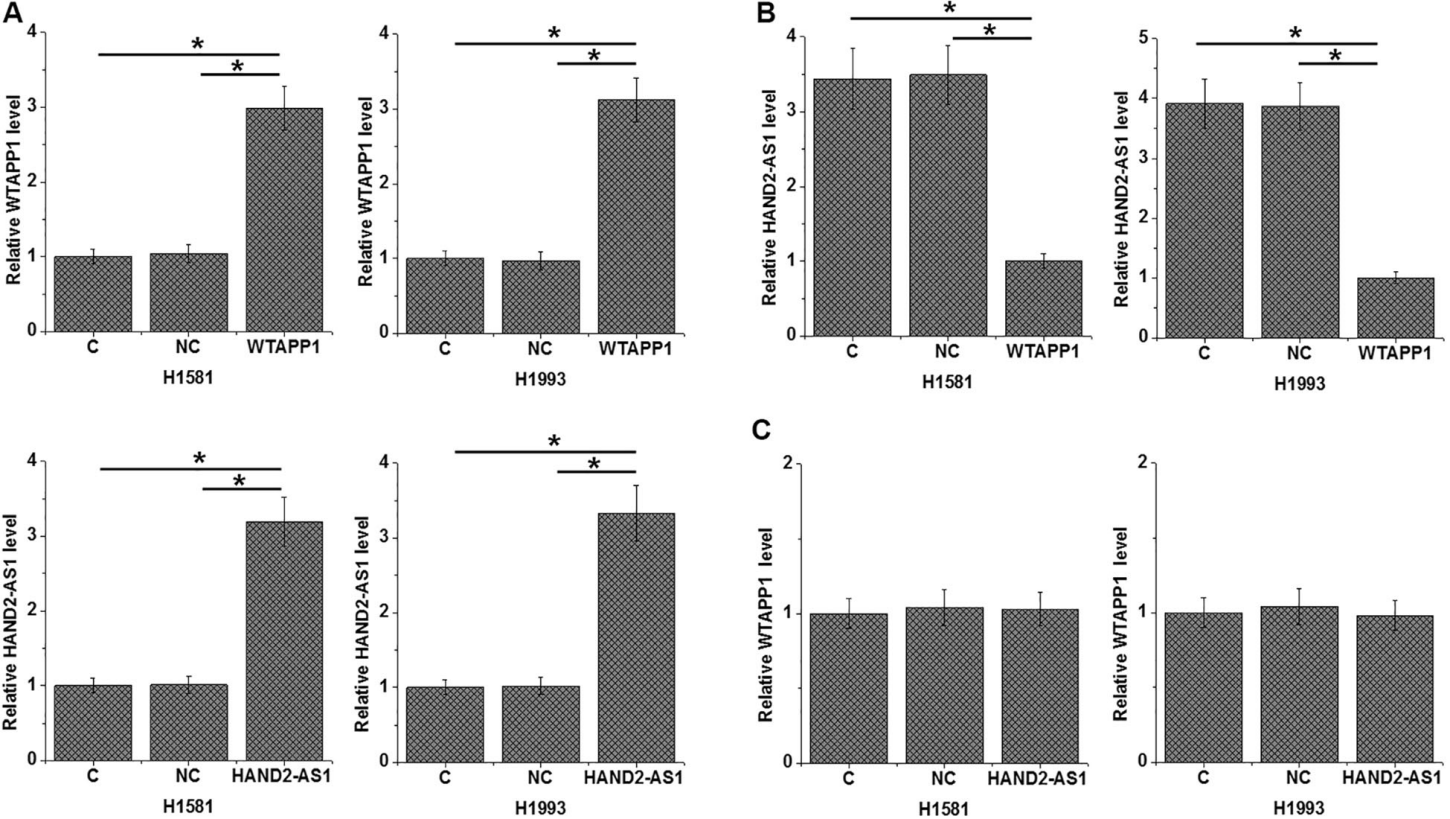
Overexpression of WTAPP1 mediated the downregulation of HAND2-AS1 in NSCLC cells. Overexpression of WTAPP1 and HAND2-AS1 were reached at 24 h after transfection **a**. Overexpression of WTAPP1 resulted in downregulation of HAND2-AS1 in NSCLC cells **b**, while overexpression of HAND2-AS1 did not affect the expression of WTAPP1 **c**

Overexpression of WTAPP1 and HAND2-AS1 vectors were transfected into cells of the two NSCLC cell lines H1581 and H1993. Comparing to C (Control) and NC (negative control) cells, overexpression of WTAPP1 and HAND2-AS1 were achieved at 24 h after transfection (Fig. 3a, *p* < 0.05). In addition, downregulation of HAND2-AS1 was observed after the overexpression of WTAPP1 in NSCLC cells (Fig. 3b, *p* < 0.05), while overexpression of HAND2-AS1 did not affect the expression of WTAPP1 (Fig. 3c).

### Overexpression of WTAPP1 led to altered NSCLC cell invasion and migration through HAND2-AS1

**Fig. 4:**
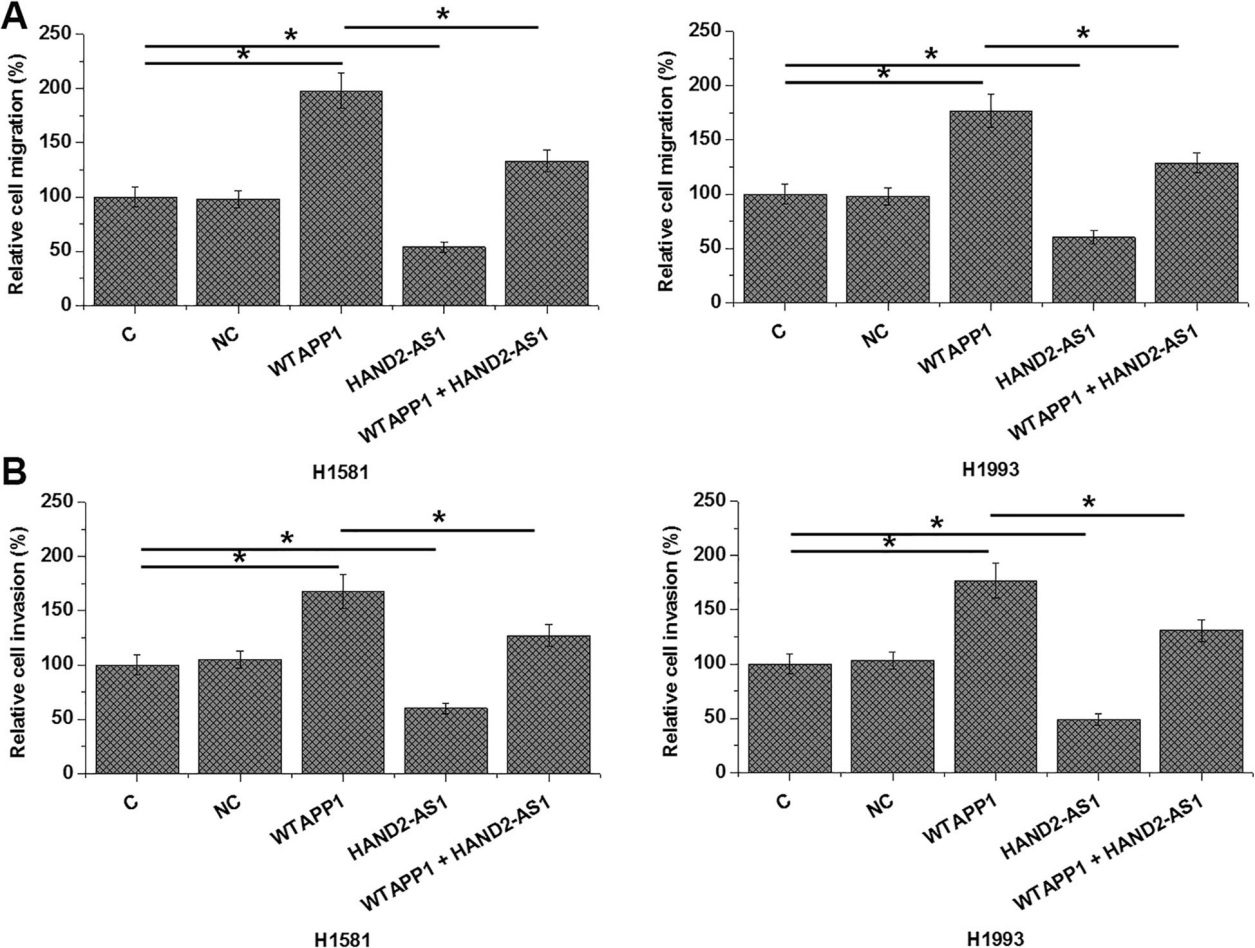
Overexpression of WTAPP1 led to altered NSCLC cell migration and invasion through HAND2-AS1 Overexpression of WTAPP1 led to promoted, while overexpression of HAND2-AS1 led to inhibited migration **a** and invasion **b** of NSCLC cells. In addition, overexpression of HAND2-AS1 partially attenuated the effects of overexpression of WTAPP1 (*, *p* < 0.05).

Comparing to C (Control) and NC (negative control) cells, overexpression of WTAPP1 led to promoted, while overexpression of HAND2-AS1 led to inhibited migration (Fig. 4a) and invasion (Fig. 4b) of NSCLC cells (*p* < 0.05). Moreover, overexpression of HAND2-AS1 partially reduced the effects of overexpression of WTAPP1 (*p* < 0.05). In addition, overexpression of WTAPP1 showed no significant effects on cancer cell proliferation (data not shown).

## Discussion

It has been reported that WTAPP1 can promote angiogenesis and migration of endothelial progenitor cells [10], while its functionality in cancer biology is unknown. We reported that WTAPP1 was upregulated in NSCLC, and WTAPP1 may serve as an oncogene in NSCLC by promoting cancer cell invasion and migration through the downregulation of HAND2-AS1.

HAND2-AS1 plays a tumor suppression role in different types of cancer [12–14]. HAND2-AS1 suppresses cancer development through the interaction with multiple downstream pathways, such as energy metabolism and TGF-β signaling (Miao et al. 2018; Kang et al. 2018). HAND2-AS1 was downregulated during the development of NSCLC, and overexpression of HAND2-AS1 resulted in inhibited cancer cell invasion and migration [13]. Our study also demonstrated the downregulation of HAND2-AS1 in NSCLC, and overexpression of HAND2-AS1 led to inhibited invasion and migration of NSCLC cells. Our data further confirmed the tumor suppression role of HAND2-AS1 in NSCLC.

LncRNAs participate in cancer biology mainly through the regulation of downstream tumor suppressive or oncogenic pathways [15]. Interestingly, our study illustrated that WTAPP1 participates in NSCLC by serving as the upstream inhibitor of another lncRNA, HAND2-AS1. However, overexpression of HAND2-AS1 only partially attenuated the enhancing effect of overexpression of WTAPP1 on NSCLC cell migration and invasion. Therefore, WTAPP1 may interact with multiple downstream effectors to achieve the regulation of NSCLC cell invasion and migration. In addition, WTAPP1 and HAND2-AS1 were not correlated in adjacent healthy tissues, indicating that WTAPP1 may not regulate the expression of HAND2-AS1 under physical conditions.

Different from messenger RNAs, lncRNAs are usually expressed in certain types of cell [16]. However, lncRNAs can also be released into circulating system from the site of synthesis to achieve systemic trafficking and regulation of gene expression [17]. However, our study did not detect WTAPP1 in plasma of NSCLC patients (data). Therefore, plasma WTAPP1 may be under detectable level or WTAPP1 may not be a systemic trafficking lncRNAs.

In conclusion, WTAPP1 is upregulated in NSCLC and promotes NSCLC by downregulating HAND2-AS1 to promote cancer cell migration and invasion.

## Data Availability

The analyzed data sets generated during the study are available from the corresponding author on reasonable request.

## Abbreviations

NSCLC: Non-small cell lung cancer
lncRNA: Long non-coding RNA
WTAPP1: Wilms Tumor 1 Associated Protein Pseudogene 1
HAND2-AS1: HAND2 Antisense RNA 1
FBS: Fetal bovine serum
